# β3 Integrin in Cardiac Fibroblast Is Critical for Extracellular Matrix Accumulation during Pressure Overload Hypertrophy in Mouse

**DOI:** 10.1371/journal.pone.0045076

**Published:** 2012-09-12

**Authors:** Sundaravadivel Balasubramanian, Lakeya Quinones, Harinath Kasiganesan, Yuhua Zhang, Dorea L. Pleasant, Kamala P. Sundararaj, Michael R. Zile, Amy D. Bradshaw, Dhandapani Kuppuswamy

**Affiliations:** 1 Cardiology Division, Department of Medicine, Gazes Cardiac Research Institute, Medical University of South Carolina, Charleston, South Carolina, United States of America; 2 Ralph H. Johnson Department of Veterans Affairs Medical Center, Charleston, South Carolina, United States of America; NCMLS, Radboud University Nijmegen Medical Center, The Netherlands

## Abstract

The adhesion receptor β3 integrin regulates diverse cellular functions in various tissues. As β3 integrin has been implicated in extracellular matrix (ECM) remodeling, we sought to explore the role of β3 integrin in cardiac fibrosis by using wild type (WT) and β3 integrin null (β3−/−) mice for *in vivo* pressure overload (PO) and *in vitro* primary cardiac fibroblast phenotypic studies. Compared to WT mice, β3−/− mice upon pressure overload hypertrophy for 4 wk by transverse aortic constriction (TAC) showed a substantially reduced accumulation of interstitial fibronectin and collagen. Moreover, pressure overloaded LV from β3−/− mice exhibited reduced levels of both fibroblast proliferation and fibroblast-specific protein-1 (FSP1) expression in early time points of PO. To test if the observed impairment of ECM accumulation in β3−/− mice was due to compromised cardiac fibroblast function, we analyzed primary cardiac fibroblasts from WT and β3−/− mice for adhesion to ECM proteins, cell spreading, proliferation, and migration in response to platelet derived growth factor-BB (PDGF, a growth factor known to promote fibrosis) stimulation. Our results showed that β3−/− cardiac fibroblasts exhibited a significant reduction in cell-matrix adhesion, cell spreading, proliferation and migration. In addition, the activation of PDGF receptor associated tyrosine kinase and non-receptor tyrosine kinase Pyk2, upon PDGF stimulation were impaired in β3−/− cells. Adenoviral expression of a dominant negative form of Pyk2 (Y402F) resulted in reduced accumulation of fibronectin. These results indicate that β3 integrin-mediated Pyk2 signaling in cardiac fibroblasts plays a critical role in PO-induced cardiac fibrosis.

## Introduction

Integrins are cell surface glycoproteins involved in a variety of cellular functions including migration, adhesion, spreading, proliferation, transcription and translation. Integrins function as a heterodimer composed of an α and a β chain. There are 8 different α chains and 18 different β chains which by multiple combinations of dimerization make at least 24 different α/β heterodimers [1]. Of the several integrin β chains expressed in the adult myocardium, both β1 and β3 have been shown to be important during cardiac stress: for instance, pressure overload of the myocardium induces both β1 and β3-mediated focal adhesion complex formation involving non-receptor tyrosine kinases such as Src and Fak in the adult cardiomyocytes [2], [3].

The activation of integrin signaling is important in the context of adult cardiomyocytes for cytoskeletal rearrangement, especially at the level of sarcomeric Z-lines as well as costameres [4]. In PO myocardium, others and we have documented integrin-mediated survival signaling via NFκB activation [5], cytoskeletal rearrangement via focal adhesion complex formation [6], [7] and cell growth via S6K activation [8], [9], [10], [11]. While these studies point to a direct role of integrins on cardiomyocytes, their role in cardiac fibroblasts, the other major cell type in the myocardium, has not been extensively studied. Cardiac fibroblasts are important for the synthesis, secretion, assembly and turnover of extracellular matrix (ECM) proteins in the myocardium for tissue homeostasis. Under pathological conditions such as PO during hypertension, ischemia/reperfusion and myocardial infarction, the myocardial cardiac fibroblasts proliferate and become a primary source of fibrotic ECM proteins [12]. Recently β1 integrin and the downstream activation of FAK have been shown to be involved in cardiac fibrosis during PO [13]. Using β3−/− mice, we previously reported that β3 integrin is important for the survival of cardiomyocytes upon induction of ventricular PO by transverse aortic constriction (TAC). These mice upon TAC exhibited a reduced myocyte cross sectional area, reduced LV ejection fraction and increased end diastolic dimension [5]. Our subsequent analysis showed a high degree of myocardial calpain activation and apoptosis in the β3−/− PO mice [14].

Based on functional analyses of β3 integrin in various cell types [15], we hypothesized that cardiac fibroblasts from β3−/− mice would also have impaired activity that would lead to a reduction in ECM accumulation in the myocardium of PO β3−/− mice. To test this idea, we analyzed whether the β3−/− mice exhibit reduced ECM accumulation upon PO and whether cardiac fibroblasts from β3−/− mice exhibited changes in integrin-dependent functions such as adhesion, migration, proliferation and ECM production versus that of WT cells. Our data suggest that β3 integrin is critical for cardiac fibroblast function under normal and PO conditions for mediating Pyk2 signaling.

## Materials and Methods

### Mice

12 wk old male C57BL/6 mice (wild type, WT) and β3−/− mice, generated in Dr. Hynes laboratory [16] were obtained from Jackson Laboratories and used for surgical and cell isolation procedures. Mice colonies were maintained at the Medical University of South Carolina (MUSC) animal care facility and all animal studies were conducted in accordance with the Guide for the Care and Use of Laboratory Animals (National Research Council, National Academy Press, Washington, DC, 1996) and were approved by the Institutional Animal Care and Use Committee at MUSC (Approval ID: ACORP443).

### Transverse Aortic Constriction (TAC)

Pressure overload was induced in age-matched WT and β3−/− mice as reported previously for 72 h, 1 wk and 4 wk [5] by tying a suture around the transverse aorta over a 27-gauge blunted needle causing occlusion of the aorta. The needle was withdrawn, resulting in a stenotic aortic lumen. Since the mortality after TAC in β3−/− mice was higher than in the WT mice [5] the upper limit for TAC duration was kept at 4 wk in the present study. While the initial few days after TAC is known to exhibit myocardial cell proliferation, the 4 wk duration is shown to be sufficient for studying ECM accumulation [17]. Therefore, we set our time points as 72 h, 1 wk and 4 wk for TAC. After the indicated TAC duration, animals were euthanized by removal of the heart in deep anesthesia. Sham-operated mice without TAC served as controls. Hemodynamic parameters and myocardial function studies showed that PO in β3−/−mice, when compared to WT mice, caused a significant increase in cardiomyocyte loss, accompanied by reduced systolic function, ejection fraction and increased left ventricular end-diastolic volume [5].

### Cardiac Fibroblast Primary Culture

Primary cardiac fibroblasts were isolated from age-matched (12–16 wk old) WT and β3−/− mice as reported previously [18]. Briefly, hearts were removed, rinsed in PBS, minced, and subjected to collagenase digestion in 1∶10 diluted Blendzyme-3 (Roche, Indianapolis, IN) in DMEM (Invitrogen) at 37°C for 1–3 h. Tissue was triturated, and the resulting cell suspensions were rinsed three times in growth media (DMEM containing 10% FBS and antibiotic-antimycotic solution) before final plating. All experiments with cardiac fibroblasts were performed between passages 2 and 3.

### Immunohistochemistry

At the end of the surgical procedure, left ventricular tissue was removed and fixed in 4% paraformaldehyde for 2 h at room temperature followed by washing in PBS for subsequent paraffin embedding or freshly frozen in OCT at −80°C. For collagen volume fraction (CVF), hearts were fixed in formalin and stained with picrosirius red as described previously [19]. CVF was determined in five fields from at least six separate animals from each group. For immunostaining, fresh frozen sections were permeabilized with 0.1% Triton X-100 and then blocked with 10% normal donkey serum for 1 h at room temperature. The sections were then incubated with primary antibodies (1∶500 dilutions) for overnight at 4°C. Following three washes in PBS, secondary antibodies conjugated to Alexa Fluor dyes (1∶1000 dilutions) and DAPI (1∶1000 dilution for nuclear staining) were incubated for 2 h at room temperature. The slides were then washed three times in PBS, mounted with coverslips using Mowiol and subjected to laser scanning confocal microscopy (LSM 501, Olympus IX71 and Olympus IX81).

### Immunocytochemistry

Cardiac fibroblasts grown on coverslips after specified treatments were fixed with 2% paraformaldehyde for 5 min, permeabilized with 0.1% Triton X-100 for 5 min and then blocked with 10% normal donkey serum for 1 h at room temperature. The primary antibodies (1∶500 dilutions) were then incubated with the sections for overnight at 4°C. Following three washes in PBS, secondary antibodies conjugated to Alexa Fluor dyes (Invitrogen; 1∶1000 dilutions) and DAPI (1∶1000 dilution) were incubated for 2 h at room temperature. The coverslips were then washed three times in PBS, mounted onto glass slides using Mowiol and subjected to laser scanning confocal microscopy (LSM 501, Olympus IX71 and Olympus IX81).

### Western Blotting

Cells or ventricular tissue samples were extracted using Triton X-100 containing buffer and processed with SDS sample buffer as described previously [5]. Proteins in SDS sample buffer were resolved by SDS-PAGE and transferred to PVDF membranes. The membranes were blocked for 1 h using 1% BSA and 5% milk in TBST (10 mM Tris, 0.1 M NaCl, 0.1% Tween-20, pH 7.4). Blots were incubated with primary antibodies in TBST overnight at 4°C, washed five times, each for five minutes with TBST, and then incubated with horseradish peroxidase conjugated secondary antibody in TBST for 1 h at room temperature. After five washes, each for 5 minutes with TBST, the proteins were detected by enhanced chemiluminescence (PerkinElmer, Wellesley, MA).

### Cell Migration and Invasion Assays

Cardiac fibroblasts migration was measured by using Oris Cell Migration Assay kit as per the manufacturer’s instructions (Platypus Technology). Briefly, WT and β3−/− cells were seeded at the same density in Oris TM 96 well plates coated with fibronectin (6 wells per condition) and allowed to grow overnight. The presence of a circular stopper in the middle of the plate prevents attachment of cells to the center during seeding. After overnight cell attachment, the stopper was removed to expose the central empty circle, which has now created a space for the surrounding cells to migrate into. The cells were stimulated to migrate by adding PDGF (10 ng/mL). After 28 h, the cells were fixed with 4% paraformaldehyde and stained with vinculin antibody (Neomarker), phalloidin-Alexa Fluor 568 (Invitrogen; for actin) and with DAPI (for nucleus) and then analyzed by fluorescence microscopy at 10X (Olympus IX71). Transwell invasion assay was performed as described elsewhere [20]. Briefly, cardiac fibroblasts from WT and β3−/− were serum starved for 16 hours and then seeded at a density of 4×10^5^ cells onto each well of 24-well Transwell inserts with a membrane pore size of 8-µm (BD Biosciences) in 0.1% FBS medium. To block cell proliferation, mitomycin C (5 µg/ml) (Sigma) was added at the time of seeding. Cells were incubated for 16 h at 37°C and fixed with 1% paraformaldehyde and stained with DAPI. The number of nuclei on the bottom of the Transwell inserts were counted using Olympus IX81 confocal microscope using a 10X objective. At least 10 random fields were recorded to count the total number of nuclei in each case and expressed as a mean (± SEM).

### Proliferation Assay

^3^H-thymidine incorporation assays were performed as reported early [21]. WT and β3−/− cardiac fibroblasts were plated at equal densities in 24-well plates (6 wells per condition) and allowed to adhere overnight. The cells were stimulated by the addition of PDGF (10 ng/mL) for 18 h and then incubated with 2 µCi/mL ^3^H-thymidine (6.7 Ci/mmol; Amersham, Arlington Heights, IL) for 4 h. The following protocol was used to measure ^3^H-thymidine incorporation: cells were 1) rinsed twice in cold PBS, 2) added with 10% trichloroacetic acid for 30 min at 4°C, 3) washed in cold 100% ethanol, 4) solubilized in 0.1 N NaOH for 30 min at 65°C, and 5) radioactivity measured by a scintillation counter. For measuring cell proliferation in the presence or absence of integrin function-blocking antibodies, cardiac fibroblasts grown in glass coverslips in 12-well plates in 10% FBS medium were incubated with respective antibodies (β3 integrin F11 antibody #554951, BD Bioscience and β1 integrin monoclonal antibody #2252, Millipore) or IgG control antibody (50 µg/mL) for 36 hours. After the incubation period, the cells were fixed and then stained for Ki67 and nucleus for confocal microscopy. The number of Ki67 positive nuclei was counted from 10 random fields under 20X and the average ± SEM from three experiments is presented as graph.

### Cell Adhesion and Spreading

WT and β3−/− cardiac fibroblasts cultured in complete medium were washed and trypsinized. The trypsinized cells were resuspended in complete medium and were allowed to readhere for 10, 30 and 120 min on plates previously coated with either fibronectin (10 µg/mL) or laminin (10 µg/mL). The cells were then fixed and stained for actin and vinculin [22]. For adhesion measurement, the number of adhered cells at each time point was counted in ten random fields and the percentage of adhered cells were then calculated and plotted. In the case of spreading, the cells displaying vinculin staining that extended out of the cortical actin staining were counted as spread cells. At least 400 cells were counted in each case to calculate the percentage of spreading in each experiment.

## Results

### Reduced Fibrotic Protein Accumulation in TAC Pressure Overloaded β3−/− Mice

**Figure 1:**
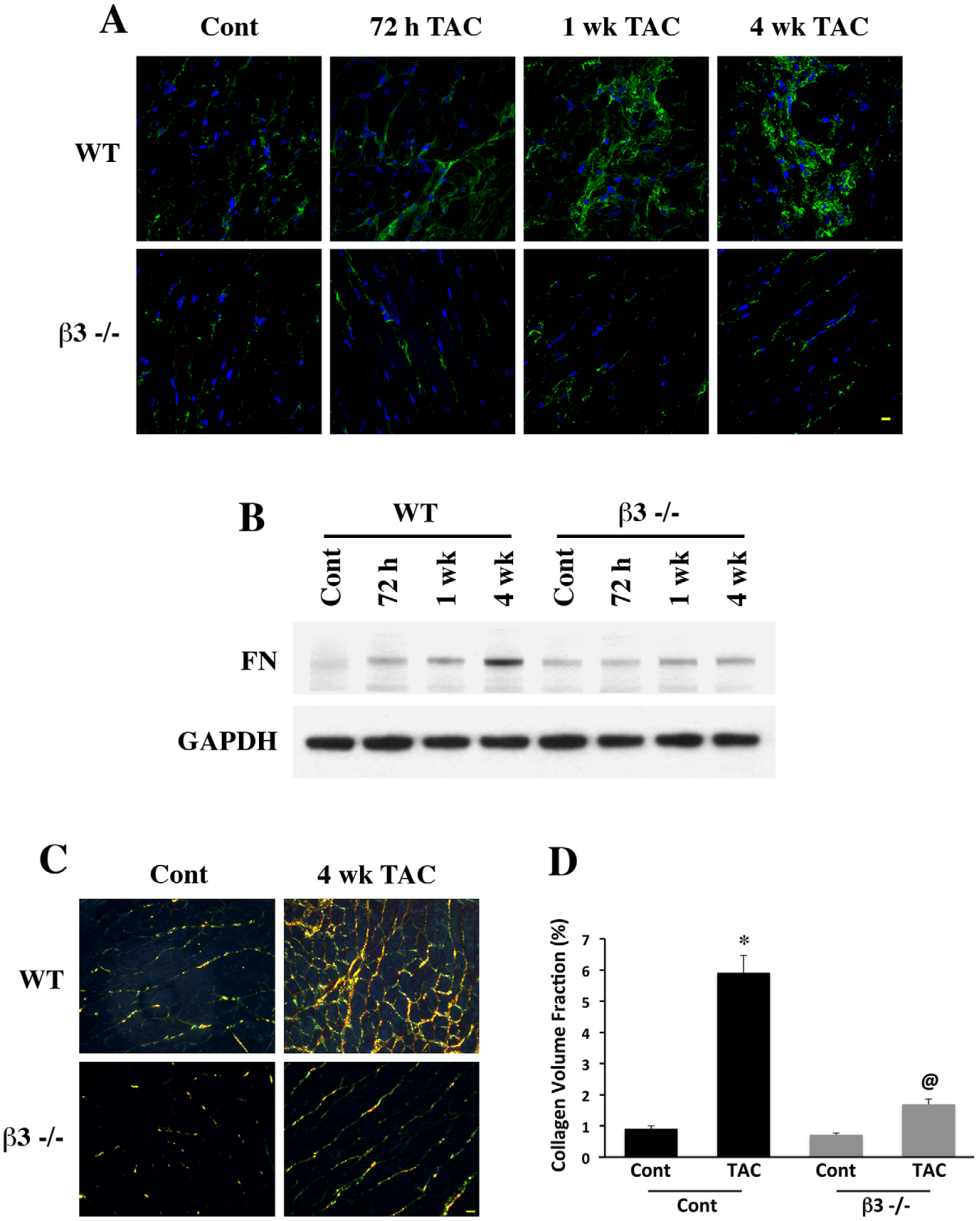
Reduction of pressure overload induced fibronectin and collagen accumulation in β3−/− mice. (**A**) WT and β3−/− mice underwent pressure overload by TAC for 72 h, 1 wk and 4 wk and LV sections were stained for fibronectin using anti-fibronectin antibody (green). LV samples from Sham operated mice served as controls. Nuclei are shown in blue (DAPI). Results were confirmed in two additional mice samples. *Scale bar, 10 µm.* (**B**) Triton-X-100 soluble fractions from the pressure-overloaded LV tissue from WT and β3−/− mice for indicated durations as shown in (A) were processed for Western blotting with fibronectin (FN) antibody. Normalized protein loading is shown with GAPDH immunoblot. This experiment was confirmed with a minimum three sets of samples. (**C**) LV tissue sections from WT and β3−/− mice with and without TAC for 4 wk were stained for collagen with picrosirius red stain. (**D**) Collagen volume fraction was calculated in photomicrographs using Sigma Scan Pro-5. Bar shows 10 µm. The numbers of mice were: WT control (n = 7); WT TAC (n = 6); β3−/− Control (n = 6); β3−/− TAC (n = 6). Individual and grouped data are presented in the graph. * p<0.05 vs. WT control; @ p<0.05 vs. WT 72 h TAC.

As a first step to analyze the involvement of β3 integrin in the fibrotic events during hypertrophy, we measured fibrotic protein accumulation following TAC for 72 h, 1 wk and 4 wk in WT and β3−/− mice along with respective sham controls. We measured two key extracellular matrix proteins, fibronectin and collagen which are induced upon integrin mediated signaling and is critical during fibrotic events after 72 h, 1 wk and 4 wk of pressure overload induction. As can be seen from Figure 1A, in all time points after TAC, there was a marked increase in fibronectin in pressure overloaded WT mice which was markedly reduced in β3−/− myocardium. The confocal data was confirmed biochemically using Western blotting (Figure 1 B) in LV tissue samples from WT and β3−/− mice after TAC for indicated durations. Since earlier studies [17] and our fibronectin data suggest that ECM secretion reaches a maximum by 4 wk, we measured collagen volume fraction at this time point. Figure 1C and 1D show the staining and the quantification of the collagen volume fraction from WT and β3−/− mice with and without TAC hypertrophy. It is evident that in the sham control animals from both the groups, the amount of interstitial collagen was low and there was not a significant difference between these two groups. However, upon 4 wk TAC induced pressure overload, there is a marked increase in collagen accumulation in the case of WT myocardium whereas the accumulation of collagen was significantly reduced in β3−/− myocardium. Quantification of collagen staining from these PO ventricular sections confirmed that there is a significant difference between WT and β3−/− groups in terms of collagen accumulation. Previous studies suggest that a 4 wk time point is ideal to evaluate cardiac fibrosis [17]. However, in the case of β3−/− mice, extending PO beyond 4 wk might be useful to know if the loss of PO-induced cardiac fibrosis in β3−/− mice was due to a delay in the synthesis and/or deposition of ECM proteins. Unfortunately, the survival of β3−/− mice after 4 wk PO was very low as we published earlier [5] and therefore we could not obtain sufficient number of mice beyond 4 wk. Nevertheless, these experiments provide the first line of evidence that β3 integrin engagement is critical for ECM accumulation during hypertrophic stimulation.

### Cardiac Fibroblast Proliferation is reduced in β3−/− Mice during PO in vivo

**Figure 2:**
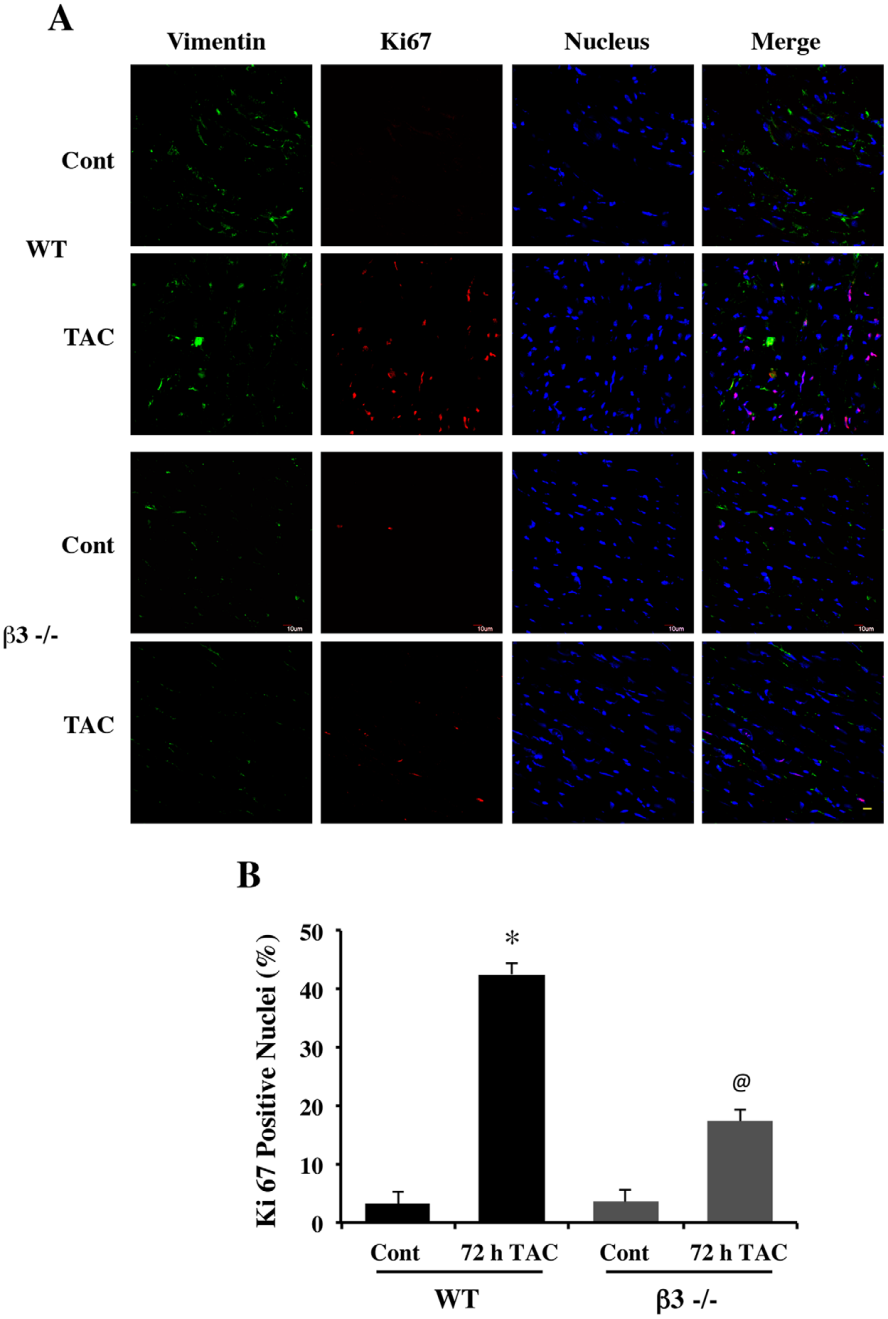
Loss of pressure overload-induced proliferation of cardiac fibroblasts in β3−/− mice. (**A**) LV tissue sections from WT and β3−/− Sham (Cont) and 72 h TAC were stained for vimentin (green), Ki67 (red), and nuclei (blue). The merged image of all three stains is also shown. *Scale bar, 20 µm.* (**B**) Quantification of the number of Ki67 positive cells in 3–5 sections from three independent mice is shown in the graph. * p<0.05 vs. WT control; @ p<0.05 vs. WT 72 h TAC.

The reduced fibronectin and collagen accumulation observed in PO β3−/− heart could be a result from decreased proliferation of cardiac fibroblasts in the myocardium. Therefore, we measured fibroblast proliferation in PO myocardium. For these *in vivo* studies, we stained LV tissue sections from control and PO WT and β3−/− mice for the proliferation marker Ki67 along with vimentin, a fibroblast marker. The confocal micrographs shown in Figure 2A indicate that the Ki67 is enriched in 72 h PO WT myocardium which was significantly reduced in the corresponding β3−/− mice. The levels of vimentin were also low in β3−/− mice. Quantification of Ki67 positive cells in 72 h TAC myocardium from both WT and β3−/− mice confirmed a significant decrease in proliferating fibroblasts in PO β3−/− mice (Figure 2B).

**Figure 3:**
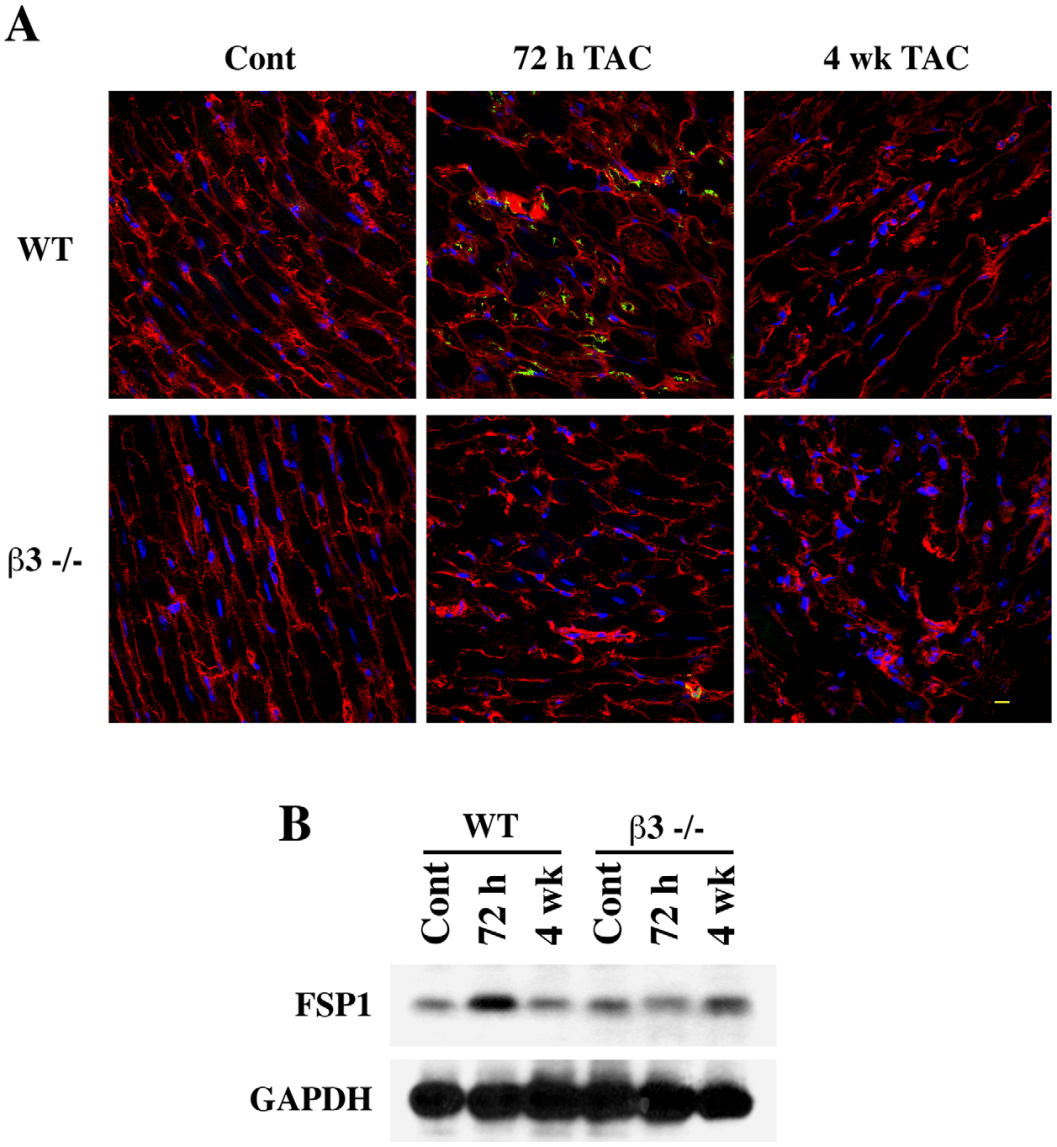
Loss of fibroblast specific protein-1 (FSP-1) expression in pressure overloaded myocardium of β3−/− mice. (**A**) WT and β3−/− mice underwent Sham (Cont) or TAC pressure overload for 72 h, or for 4 wk. Tissue sections were stained for FSP-1 (green) and vinculin (red) with specific antibodies. (Nuclei stained with DAPI, blue). The merged images of all three stains are shown. *Scale bar, 10 µm.* (**B**) Western blot analysis for Triton X-100 soluble LV tissue samples from mice groups as shown in (A) above were analyzed for the presence of FSP1 (top) and GAPDH (bottom) with specific antibodies.

We measured the expression of another independent marker to further evaluate cardiac fibroblast proliferation. FSP1 (fibroblast specific protein-1) is a nonmuscle cell marker that is highly expressed in actively proliferating fibroblasts. In hypertrophic myocardium, since the expression of FSP1 could be induced in the first few days upon hypertrophic induction and could last up to several weeks [23], we chose an early (72 h) and a late (4 wk) time points to study FSP1 expression. As can be seen from Figure 3A, whereas WT mice upon 72 h of TAC exhibited a marked elevation of FSP1 in LV when compared to sham control mice, the β3−/− mice LV did not show such an induction of FSP1 upon TAC. The costameric localization of vinculin was prominent in WT tissue whereas it was disarrayed in β3−/− mice tissue under PO conditions. The induction of FSP1 expression upon 72 h TAC in WT but not in β3−/− mice was confirmed with Western blotting as well (Figure 3B). These *in vivo* studies demonstrate that fibroblast proliferation accompanied by fibrotic protein expression is substantially reduced in response to PO hypertrophy in the absence of β3 integrin.

### Compromised Cell Proliferation, Migration, Cell Adhesion and Spreading of Isolated Primary Cardiac Fibroblasts from β3−/− Mice

**Figure 4:**
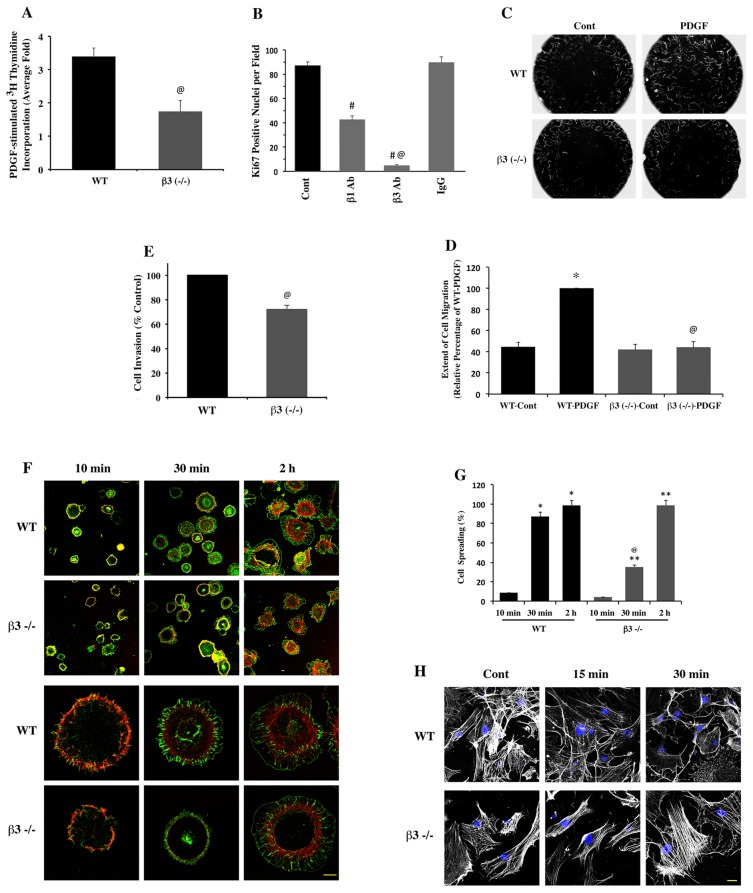
Phenotypic characteristics of isolated cardiac fibroblasts from β3−/− mice. (**A**) WT and β3−/− cardiac fibroblasts were subjected to ^3^H-thymidine incorporation assay as described in Materials and Methods. Fold-induction in PDGF stimulated ^3^H-thymidine incorporation was calculated between WT control and WT+PDGF, and β3−/− control and β3−/− + PDGF. The difference in fold induction of cell proliferation between WT and β3−/− obtained from 5 independent experiments is presented as average ± SEM. @ p<0.005 vs. WT. (**B**) For comparing the contribution of β3 and β1 integrins using function blocking antibodies, WT cardiac fibroblasts were plated on glass coverslips in DMEM containing 10% FBS. After overnight incubation, the cells were incubated with indicated function blocking or control antibodies (50 µg/mL) for 36 hours. After the incubation period, the cells were fixed and stained for Ki67 and nucleus. The number of Ki67 positive nuclei was counted from 10 random fields of confocal images under 20X objective and the average ± SEM presented as a graph. # p<0.05 vs. control; @ p<0.05 vs. control and β1 Ab. (**C**) For the cell migration assay, WT and β3−/− cells were plated in 96 well format Oris TM plates (Platypus Technology) and allowed to grow overnight. Stoppers were then removed and the cells were stimulated to migrate by adding PDGF (10 ng/mL). After 28 h, the cells were fixed with 4% paraformaldehyde and stained for actin (phalloidin-Alexa Fluor 568; red) and then the entire well was imaged by fluorescent microscopy at 10X. (**D**) Quantification from the migration assay is depicted in the graph. *p<0.05 vs. WT control; @ p<0.05 vs. WT-PDGF. (**E**) For Transwell cell invasion assay, WT and β3−/− cardiac fibroblasts were seeded onto Transwell inserts in 0.1% FBS medium in the presence of mitomycin C (5 µg/ml). Cells were incubated for 16 h at 37°C in the presence of PDGF (10 ng/ml) at the bottom chamber and fixed with 1% paraformaldehyde and stained with DAPI. The number of nuclei on the bottom of the Transwell inserts was counted using a 10X objective. At least 10 random fields were recorded to count the total number of nuclei in each case and expressed as a mean (± SEM). @ p<0.05 vs. WT. (**F**) For the adhesion experiments, WT and β3−/− cardiac fibroblasts cultured in complete medium were washed and trypsinized. The suspended cells in complete medium were re-adhered on fibronectin-coated plates for 10 min, 30 min and 2 h. The cells were then fixed and stained for actin (red) and vinculin (green). Lower magnification photomicrograph is shown in (Top panel) and the higher magnification photomicrograph is shown in (Bottom panel). *Scale bar, 10 µm*. (**G**) For cell spreading measurements, cardiac fibroblasts from WT and β3−/− mice were trypsinized, plated on fibronectin-coated plates for 10 min, 30 min and 2 h. After washing away the non-adherent cells, the adhered cells were fixed and stained for actin (Phalloidin-Alexa Fluor 568) and vinculin. The cells were imaged using Olympus (IX71). Cells that showed vinculin staining that reached out of the actin ring/cell body were considered spread cells. At least 400 cells were measured in each case to calculate the % of spreading in each experiment. * p<0.05 vs. WT 10 min; ** p<0.05 vs. β3−/−10 min; @ p<0.05 vs. WT 10 min. (**H**) To analyze altered actin cytoskeletal changes in PDGF-induced cardiac fibroblasts, cells from WT and β3−/− grown on coverslips were serum starved for 16 h and then stimulated with PDGF (10 ng/mL) for indicated durations and fixed with 1% paraformaldehyde. The cells were then stained for actin (Grey) and nucleus (DAPI, blue) and imaged under confocal microscopy using 60X oil objective. *Scale bar, 10 µm*.

To further determine whether reduced fibroblast proliferation and associated reductions in ECM observed *in vivo* could be demonstrated *in vitro* using β3−/− cardiac fibroblasts, we isolated adult primary cardiac fibroblasts from WT and β3−/− mice and analyzed their response to PDGF, a growth factor overexpressed in hypertrophic myocardium and known to cause synergistic signaling with β3 integrin [13], [24]. First, cell proliferation was quantified using ^3^H-thymidine incorporation in response to PDGF stimulation. Fold differences in proliferation between WT and β3−/− cardiac fibroblasts from five independent experiments revealed a significant reduction in proliferation by β3−/− cells in response to PDGF (Figure 4A). These data suggest that β3 integrin plays a key role in the proliferation of cardiac fibroblasts under growth factor stimulation. To assess the comparative contribution of β3 and β1 integrins for cell proliferation, since the latter is the other prominent integrin subtype in the myocardium, we incubated cardiac fibroblasts from WT mice with function-blocking antibodies in 10% serum medium for 36 hours and fixed the cells and stained them with Ki67 and DAPI to quantify the number of Ki67 positive cells using confocal microscopy. As can be seen from Figure 4B even though blocking with β1 integrin antibody resulted in a significant reduction (57.4±2.9%) in the number of Ki67 positive cells, blocking with β3 integrin antibody exhibited stronger inhibition where only 4.8±0.6% cells showed Ki67 staining. Use of separate additional independent function-blocking antibodies confirmed these results. This data further confirms that β3 integrin plays a predominant role in cardiac fibroblast proliferation.

Next, β3-integrin dependent migration of cardiac fibroblasts was analyzed by adopting a relatively new method similar to that used in the wound closure assay [25]. Cells were first plated on a fibronectin coated 96 well plate where the central circle (2 mm) of the wells was covered with a stopper to avoid initial attachment of cells within this circle. After overnight attachment of cells in the peripheral surface in the well, the central area was cleared by removal of the stopper and PDGF (10 ng/mL) was added to part of the wells. Cells were allowed to migrate to the inner circle by incubating an additional 28 h (Figure 4C). The quantitative data from this experiment shown in Figure 4D suggested that there was a marked reduction in PDGF-stimulated cell migration into the central space in β3−/− cells when compared to WT cells. However, part of such reduction might be due to reduced proliferation by β3−/− cells. Therefore, we performed Transwell invasion assay in the presence of cell cycle inhibitor mitomycin (Figure 4E) as described in the Methods section. When compared to WT cells, the β3−/− cardiac fibroblasts showed 28% reduced invasion towards PDGF suggesting the requirement of β3 integrin for three-dimensional migration of fibroblasts. These data suggest that both cell migration on fibronectin-coated surface and cell invasion towards PDGF gradient require β3 integrin.

One of the major functions attributed to integrins is to promote adhesion of cells to ECM proteins. Therefore, we analyzed if cardiac fibroblasts from β3−/− mouse exhibited any differences in adhesion to ECM proteins when compared to WT cardiac fibroblasts. Suspended cells adhered onto fibronectin and fixed at 10 min, 30 min and 2 h time points showed no significant differences in the number of adhered cells between WT and β3−/− cells. However, the β3−/− cells showed considerable morphological defects at 10 min and 30 min (Figure 4F). That is, as shown by higher magnification (Figure 4F, lower panel), WT cells showed the presence of a continuous actin ring that colocalized with vinculin, whereas the β3−/− cells showed a discontinuous actin ring with disorganized vinculin staining. To evaluate this further, we measured an index of cell spreading by counting the number of cells that exhibited prominent vinculin-stained membrane protrusions (focal adhesions) oriented outwards from the cortical actin ring. This experiment revealed that at the 30 min time point, only 30% of the β3−/− cardiac fibroblasts exhibited this level of cell spreading whereas about 80% of the WT cardiac fibroblasts were positive for this well spread morphology (Figure 4G). However, data obtained 24 h after initial plating showed no significant differences between WT and β3−/− cells (data not shown). These data suggested that in the absence of β3 integrin, the cells did not spread efficiently on fibronectin. In addition, when cell adhesion studies were performed on laminin, there was also a significant reduction in the number of β3−/− cells adhered at the 10 min time point, although this difference was not seen at later time points (data not shown). These results suggested that β3−/− cells exhibited a significant adhesion defect to myocardial ECM proteins, in the initial phases of cell attachment.

Next, we determined growth factor-induced cytoskeletal rearrangement in WT and β3−/− cardiac fibroblasts after PDGF treatment for 15 and 30 min followed by 16 h of serum starvation. As seen in Figure 4H (30 min panel), the stress fibers in WT cells were rearranged into a punctate actin containing structures whereas β3−/− cells retained their stress fibers. These data indicate that β3 integrin is required for cytoskeletal rearrangement upon PDGF stimulation.

### β3−/− Cardiac Fibroblasts Exhibit reduced Activation of Select Tyrosine Kinases upon PDGF Stimulation

**Figure 5:**
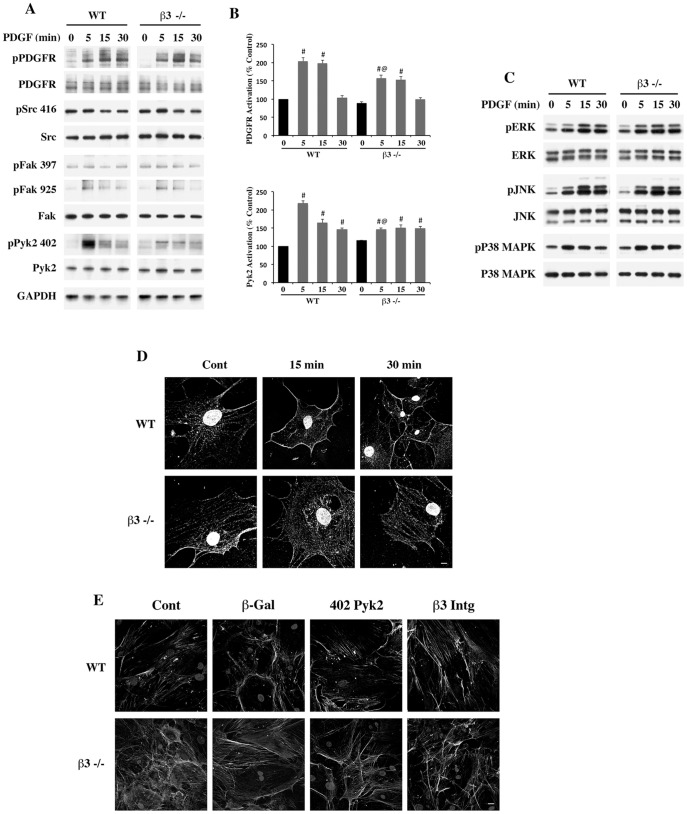
Loss of PDGF-induced activation of tyrosine kinases in isolated primary cardiac fibroblasts from β3−/− mice. (**A**) Equal numbers of WT and β3−/− cardiac fibroblasts were allowed to adhere for 2 h in 35 mm culture dishes. Cells were stimulated with PDGF (10 ng/mL) for the indicated durations following overnight serum starvation. Triton-X-100 soluble cell extracts were analyzed by Western blotting using indicated phospho-specific antibodies. Individual total proteins are shown at the bottom. Blots are representative of data from three independent experiments. (**B**) The ratio of phosphorylated to total proteins in the case of PDGFR and Pyk2 are shown as average ± SEM. # p<0.05 vs. control; @ p<0.05 vs. WT 5 min. (**C**) Cell lysates from CFb as shown in (A) above were immunoblotted with indicated phospho-specific MAPK pathway members. Individual total proteins are shown at the bottom. Blots are representative of data from three independent experiments. (**D**) WT and β3−/− cardiac fibroblasts were serum starved for 16 h and then treated with PDGF (10 ng/mL) for indicated durations and fixed with 1% paraformaldehyde. The cells were then stained with phospho-PDGFR (α-Y849/β-Y857) (Red) and phosphor-Pyk2 (Y402) (Grey) antibodies and imaged under confocal microscopy using 60X oil objective. *Scale bar, 10 µm*. (**E**) WT cardiac fibroblasts were plated on glass coverslips in DMEM containing 10% FBS. After overnight incubation, the cells were either uninfected or infected with *β-gal,* Y402F Pyk2 (402 Pyk2) or β3-integrin (β3 Intg) at an MOI of 50. After 36 h of infection, the cells were fixed and stained for fibronectin (Green) and actin (Grey) and subjected to confocal microscopy as shown under (D). Representative images were from at least three independent experiments. *Scale bar, 20 µm.*

Since integrins are upstream of key non-receptor kinases primarily Src, FAK and Pyk2 which are involved in adhesion, migration, proliferation and ECM synthesis, we determined the differential activation of these tyrosine kinases in response to PDGF stimulation by measuring the level of phosphorylated tyrosine kinases. Our data shown in Figure 5A indicates that the activation site phosphorylation of PDGFR (α-Y849/β-Y857) and Pyk2 (Y402) are remarkably reduced at all time points in β3−/− cells when compared to WT cells. In the case of FAK and Src (c-Src Y416 and FAK Y397) such differences were not significant. Since PDGF-induced activation of PDGFR and the downstream Pyk2 signaling are predominantly diminished in β3−/− fibroblasts when compared to WT fibroblasts, we quantified the ratio of phosphorylated proteins to their respective total proteins (Figure 5B). In proliferating cells, growth factors are known to stimulate MAPK signaling and therefore, we measured the activation site phosphorylation of ERK, p38 MAPK, and JNK in WT and β3−/− cardiac fibroblasts. As can be seen from Figure 5C, the PDGF-induced MAPK activation was similar in WT and β3−/− cardiac fibroblasts suggesting that these cells could use alternative integrin subtypes for MAPK activation. However, a minor decrease in p38 MAPK dephosphorylation was noted at time points 15 min and 30 min in β3−/− cardiac fibroblasts when compared to WT cardiac fibroblasts. A similar reduction in dephosphorylation was observed in PKCµ (PKD) phosphorylation (S744/748, data not shown). These data indicate that although the MAPK pathway is activated in a similar fashion in both WT and β3−/− cells, there could be differences in the desensitization of selective signaling pathways in the absence of β3 integrin.

### β3 integrin is Required for Pyk2 Cell Surface Localization and Fibronectin Accumulation

Since PDGF stimulation causes cell surface localization and activation of PDGFR and downstream nonreceptor tyrosine kinases (NTKs), we analyzed the cell surface localization of activated PDGFR and Pyk2, a kinase activated predominantly during PDGF stimulation of cardiac fibroblasts. Figure 5D shows that at 15 min after stimulation both phospho-PDGFR (α-Y849/β-Y857) and phospho-Pyk2 (Y402) showed localization to the cell membrane that subsequently exhibited a diffused pattern by 30 min. In the case of β3−/− cells, this trend in membrane localization of phospho-PDGFR and phospho-Pyk2 after PDGF treatment was not observed. These data indicate that β3 integrin is necessary for the localization of both activated PDGFR and Pyk2 during PDGF stimulation. As Pyk2 activation has been linked to ECM accumulation [26], we overexpressed kinase inactive Y402F Pyk2 by adenoviral infection and measured fibronectin deposition. As can be seen in Figure 5E, there was a dramatic reduction in extracellular fibronectin staining in cells infected with Y402F Pyk2 adenovirus when compared to controls or β-gal infected cells. It is to be noted here that extracellular fibronectin accumulation reduced in β3−/− cells could be rescued by the adenoviral-mediated expression of β3 integrin, reiterating our notion that β3 integrin mediates extracellular fibronectin accumulation. These data confirm that β3 integrin/Pyk2 pathway is critical for ECM protein accumulation in cardiac fibroblasts. Together, our data show for the first time the hypo-fibrotic phenotypes of β3−/− mice *in vivo* after TAC and in isolated β3−/− cardiac fibroblasts.

## Discussion

Cardiac fibroblasts, which account for two thirds of the total cardiac cell population [27], are the primary cell type responsible for ECM secretion, and elevated function of cardiac fibroblasts in hypertrophied heart has been shown to contribute to cardiac fibrosis [28] as well as for the associated maladaptive phenotype [27], [29], [30]. Since β3 integrin has been shown to play a critical role in cell adhesion and function of multiple cell types [15], we explored if this integrin might serve as a target to attenuate cardiac fibrosis associated with hypertensive heart diseases.

The role of β3 integrin, which is an important integrin receptor associated with cell proliferation, survival and cancer metastasis, is not well studied in the context of the cardiac fibroblasts. Dependence of integrins, especially integrin β3 for various cellular functions has been established in several cell types including cancer cells [31]. Similar to targeting β3integrin in the treatment of various cancers, it has also been suggested that β3 integrin might be a viable anti-fibrotic target in various fibrotic diseases [32]. Our study provides support to this idea as the expression of key pro-fibrotic markers in the heart during hypertrophic stimulation was found to be downregulated in the absence of β3 integrin.

In our previous studies we have shown an elevated level of programmed cell death in cardiomyocytes in 4 wk PO β3−/− myocardium, indicating that β3 integrin signaling is critical for cardiomyocyte survival [14]. The observed cardiomyocyte loss in these mice could be a combination of β3 integrin deficiency at the level of cardiomyocytes or disengagement of cardiomyocytes due to ECM insufficiency (anoikis) [33]. Although normal secretion and assembly of ECM proteins are essential for cardiomyocyte growth and survival, especially during pathological stress such as pressure overload and myocardial infraction, their excessive accumulation has been shown to contribute to tissue fibrosis and adverse remodeling [34]. Our studies using *in vivo* ventricular PO animal model, where we employed both WT and β3−/− mice, demonstrated the essential role of β3 integrin in deposition of collagen and fibronectin in PO myocardium. Our *in vivo* data suggesting a reduced ECM deposition could be due to the compounding phenotypes of germline β3−/− mice, such as bleeding disorders and reduced red blood cell numbers [35]. To understand β3 integrin function at the level of cardiac fibroblasts, we performed *in vitro* studies using isolated cardiac fibroblasts from these mice. In support of our *in vivo* findings, the *in vitro* studies using cardiac fibroblasts indicated that the loss of β3 integrin could affect ECM accumulation. Since ECM accumulation could be a cumulative result of synthesis, secretion, deposition (post-synthetic processing) and interstitial clearance by matrix metallo-proteinases (MMPs), we cannot rule out that the absence of β3 integrin in this global knockout mouse could have an effect on all or any of these processes. However, it is clear from our data that in the hypertrophied myocardium, β3 integrin is mainly responsible for accumulating collagen and fibronectin and therefore this integrin subtype could be one of the potential targets to attenuate cardiac fibrosis associated with several cardiac disease conditions. Based on earlier studies that demonstrated a critical role for β3 integrin in cell proliferation [36], the loss of PDGF-stimulated proliferation in cardiac fibroblasts from β3−/− versus WT mice confirmed that integrin co-stimulation is required for cardiac fibroblast proliferation upon PDGF growth factor stimulation. This reduction in cardiac fibroblast number might also be a contributing factor to low ECM synthesis and deposition in β3−/− mice with TAC. The importance of β3 integrin was further confirmed by our function-blocking experiments where a stronger reduction in cell proliferation was observed in the case of β3 blocking when compared with β1 function blocking.

The other activity examined here, cell adhesion and spreading, has also been shown to be a primary factor for cardiac fibroblast function as the anchoring of cells to new cellular environments is generally considered to be a rapid process. Various subtypes of integrins mediate cell adhesion in a cell type-specific manner [37]. In cardiac fibroblasts, previous studies demonstrated the involvement of β1 integrin in cell adhesion [13]. However, β3, while widely known as a primary mediator of cell adhesion and signaling in other cell types, has not been studied in cardiac fibroblasts. Our data demonstrated for the first time that β3−/− cardiac fibroblasts exhibited a slower rate of adhesion on both fibronectin and laminin. The apparent WT cell adhesion observed at later times (24 h) after plating might be due to compensatory secretion of other ECM proteins that facilitated cell adhesion via different integrin subtypes. In fact, our studies indicated that the level of β1 integrin in the hearts of these β3−/− mice was slightly higher than that of WT (data not shown). However, compensation by other integrins in β3−/− mice did not appear to be sufficient to restore ECM accumulation to that observed in *in vivo* PO myocardium. Defective adhesion in β3−/− cells has been shown to reduce adhesion to bone surfaces and thus decrease bone resorption by osteoclasts [38] further supporting our data that defective adhesion of β3−/− cardiac fibroblasts in myocardium might contribute to the hypo-fibrotic phenotype after TAC-induced hypertrophy. The defective adhesion observed in β3−/− cardiac fibroblasts was also reflected in cell spreading assays performed on fibronectin and laminin. Spreading of cardiac fibroblasts is essential for subsequent cell functions within the tissue microenvironment. Reduced β3 integrin-mediated spreading is predicted to affect the migration of fibroblasts within the interstitium and thus reduced function in the overall tissue [38], [39]. Our studies are in line with earlier studies that showed that β3 integrins were important determinants for cell spreading, in fact, impaired spreading might be another determinant for the reduced fibrosis in β3−/− mice under PO condition. However, it should be noted here that β3−/− cells exhibited a normal spreading morphology after 24 h of attachment, which suggested that other integrin subtypes on cardiac fibroblasts, most likely β [13], compensated for this spreading function.

Migration of cardiac fibroblasts within the myocardial interstitium facilitates tissue homeostasis under normal conditions and during tissue repair. Integrins contribute primarily to this process in virtually all types of cells. Cardiac fibroblasts isolated from PO myocardium exhibited significant changes in various phenotypes including collagen gel contraction, migration and proliferation within 7 days of pressure overload [40]. Various studies using cancer cells demonstrated that metastatic cells overexpressed β3 integrin and exhibited a hyper-migratory phenotype. In fact, antagonists of αvβ3 integrins are used as anti-cancer drugs, e.g., abegrin and are shown to hamper cancer cell proliferation and migration [41]. Our data showing impairment in not only the two-dimensional migration but also three-dimensional invasion in β3−/− cardiac fibroblasts upon PDGF stimulation implies that cardiac fibroblasts might exhibit a reduced migration in addition to a reduction in the number, adhesion to ECM and spreading, and thus contribute to a overall reduction in the local production of ECM proteins in the *in vivo* conditions also. In support of our findings, the anti-fibrotic effects of B-type natriuetic peptide (BNP) have been shown to effect binding of RGD-containing ECM proteins to integrin αvβ3, thus blocking β3 integrin signaling [42]. Defect in adhesion, spreading and migration could be linked to impaired ability of cytoskeletal rearrangement by β3−/− cells as seen in the present study. While it has been reported that β3 integrins mediates cytoskeletal rearrangements and supports a persistent lamellipodial mode of migration in other cells [43], the absence of such a rearrangement in β3−/− cells indicate that this integrin subtype is necessary for the activation of downstream Rho family GTPases and cytoskeletal rearrangement.

In addition to its role in cytoskeletal organization, β3 integrin plays an important role in cellular signaling. The cytoplasmic domain of β3 integrin forms multi-protein complexes (focal adhesion complex) with several adapter molecules and kinases. These kinases are primarily NTKs such as c-Src, Pyk2 and FAK, which play critical roles in numerous cellular functions, including cell survival, migration and proliferation [44]. Our studies showed a minor reduction in c-Src activation in β3−/− cells indicating that this integrin subtype is likely to mediate PDGF induced costimulation of downstream signaling. As mentioned earlier, the synergistic activation of β3 integrin and PDGFR is important for NTK activation [45]. Absence of c-Src in osteoclasts resulted in impaired migration and bone resorption and reduced actin cytoskeletal remodeling [46], [47] indicating that c-Src is a key mediator of integrin-dependent cytoskeletal functions. Furthermore, in β3−/− cells the reduction in Y925 FAK phosphorylation (Figure 5), a substrate motif for phosphorylation by c-Src, further demonstrated that c-Src activity was impaired in β3−/− cells. FAK activation as determined via its Y397 phosphorylation has been shown to mediate cyclic stretch induced differentiation of myofibroblasts [48]. However, we found that the FAK autophosphorylation site Y397 was not differentially affected in β3−/− cells indicating that this kinase might be activated by other integrin subtypes expressed in cardiac fibroblasts such as α5β1, αvβ1, and αvβ5 [1]. Of the three NTKs studied, the activation of Pyk2 was found to be significantly reduced in β3−/− cardiac fibroblasts. Pyk2 is known to be activated by integrins, growth factors, G-protein coupled receptors and upon intracellular calcium release [49]. Cells deficient in Pyk2 showed a defective migration and β-actin dynamics in osteoclasts [50]. Because Pyk2 is a critical component in migratory signaling (as well as in transcriptional and translational control), Pyk2 is expected to play a significant role in cardiac fibroblast function, especially under conditions of tissue injury. Our results indicated that Pyk2 activation was markedly impaired in β3−/− cells upon PDGF stimulation, implying that β3 integrin dependent Pyk2 activation might be a critical factor in mediating fibrosis in the heart, particularly during cardiac stress. Although Pyk2 activation is linked to the activation of MAPK family members [26], our data showing a normal MAPK activation profile in PDGF-stimulated β3−/− cells suggests that members of MAPK family could be activated independent of Pyk2 during PDGF stimulation. However, our data clearly indicate that Pyk2 activation is essential for ECM secretion. Overall, our studies establish the importance of β3 integrin/Pyk2 signaling in cardiac fibroblasts of PO myocardium, suggesting this pathway as a potential anti-fibrotic target. However, care must be taken not to overtly reduce β3 integrin function, as it is important for maintaining normal tissue homeostasis and for the survival of cardiomyocytes [6].
